# Male involvement in prevention of mother to child transmission of human immuno virus and associated factors among partners’ of reproductive age women at Debre Tabor town, Northwest Ethiopia: a community based cross sectional study

**DOI:** 10.1186/s13104-020-05023-3

**Published:** 2020-03-30

**Authors:** Enyew Dagnew, Miteku Andualem, Temesegen Worku, Dawit Gebeyehu, Wubet Taklual, Abenezer Melkie

**Affiliations:** 1 College of Health Sciences, Debre Tabor University, Post Office Box: 272, Debre Tabor, Ethiopia; 2 College of Medicine and Health Sciences, Gondar University, Gondar, Ethiopia

**Keywords:** Male involvement, PMTCT, Ethiopia, Debre Tabor

## Abstract

**Objective:**

The aim of this study was to determine the prevalence of male involvement in prevention of mother to child transmission (PMTCT) of human immune virus (HIV) and associated factors among partners’ of reproductive age women at Debre Tabor town, Northwest Ethiopia. A community based cross sectional study was employed among 561 study participants. Data was collected with pretested structured questionnaire. The data was entered by Epi-Info version 7 software and exported to SPSS version 23 for analysis. Statistical significance was declared at P value of < 0.05.

**Results:**

In this study, only 119 (21.2%) of males (95% CI 17.8%, 24.8%) were involved in PMTCT of HIV. Being government employee (AOR = 3.73, 95%CI (2.169, 6.419)), had ever heard about PMTCT of HIV (AOR = 2.46, 95%CI (1.20, 5.02)), discussed with their partner (AOR = 3.11, 95%CI (1.43, 6.55)), partners’ who were informed the need to go PMTCT of HIV clinic (AOR = 2.45, 95%CI (1.17, 5.14)), Health workers friendly approach (AOR = 2.36, 95%CI (1.34, 4.15)), and long waiting time (AOR = 0.36, 95%CI (0.216, 0.610)) were found to be significantly associated with male involvement in PMTCT of HIV. Improving service provision including respectful care and health education on PMTCT of HIV for males and their partners shall be emphasized by the government.

## Introduction

Globally male involvement is a part of woman-centered approach [1]. Worldwide males were participate in sexual and reproductive health (SRH) programs like; family planning, anti-natal care (ANC), and PMTCT of HIV program which have a great impact on the health of women, children, families and communities at large [2].

Mother-to-child transmission (MTCT) of HIV infection remains a major public health problem and more than 90% of childhood HIV infections are due to MTCT [3, 4]. Male partner co-operation in PMTCT program have been proven to be effective to reduce the risk of MTCT of HIV [5]. However, studies showed that male involvement in PMTCT of HIV is low [6]. Studies conducted in Thailand, Uganda, Kenya, Mekelle Ethiopia and Gondar Ethiopia showed that the prevalence of male involvement in the PMTCT of HIV were 46%, 5%, 31%, 20.1%, and 27.3%, respectively [7–11]. Age, residence, marital status, educational level, occupational status, average monthly income, and distance from the health institution were the significant risk factors for the PMTCT of HIV in a previous studies [12–15].

Despite, high rate of MTCT of HIV in Ethiopia, there is insufficient information regarding to males involvement in PMTCT of HIV service utilization [16]. Therefore, assessing male involvement in PMTCT of HIV is very important for program implementers and evaluators in the nation specifically in the study area. In addition, to the best of our knowledge there are paucity of studies exploring the prevalence of males involvement and associated factors in PMTCT of HIV in the study area. Thus, the aim of this study was to assess the prevalence of male involvement and its associated factors at Debre Tabor Town, Northwest, Ethiopia.

## Main text

### Methods

#### Study area, design and population

A community based cross sectional study was conducted at Debre Tabor town from Dec 2018–Feb/2019. The town is located about 667 km North of Addis Ababa (the capital city of Ethiopia) and 98 km away from Bahir Dar (the capital city of Amhara Regional State). Debre Tabor is the first industrial village in Africa with King Tewodros second at Gaffat. The town has a total population of 87,627, among these 54% were females.

The study participants were males who have reproductive age group female partner. In this study males whose reproductive age group partners gave birth during the previous 1 year or pregnant and start ANC follow up at least once were included in the study area.

#### Sample size determination

The sample size was calculated by using a single population proportion formula with 95% confidence level, 5% margin of error, 20.9% of population proportion [13], and design effect of two. The final sample size after adding 10% non-response rate was 561.

#### Sampling procedure and data collection method

Multi-stage cluster sampling technique was used. The town has four kebeles and each kebele was considered as a cluster. Simple random sampling technique was used to select clusters and we were select kebele one and three. The kebeles have been subdivided by ketsenas. Kebele one has 7 ketsenas and kebele three has five ketsenas. Finally, three ketsenas from kebele one and two ketsenas from kebele three were selected using simple random sampling technique. Systematic sampling technique with proportional allocation was used to select study participants until the desired sample size fulfill from the selected ketsenas.

Data was collected by pretested structured questionnaire through face to face interview by trained data collectors. The questionnaire used for this study was adapted from different studies [17, 18] (Additional file 1). The quality of the data was maintained during interview, entry, cleaning and analysis.

#### Data analysis

Data was entered by Epi-Info version 7 software and exported to SPSS version 23 for cleaning, editing and analysis. Bivariable and multivariable logistic regression analysis were performed to identify factors associated with male involvement on PMTCT of HIV. Variables with P value of < 0.2 in the bivariable analysis were entered in the multivariable model to identify predictors of male involvement on PMTCT of HIV. P value of < 0.05 with 95%CI was used to declare statistical significance.

#### Ethical approval

Ethical clearance was obtained from research review committee of University of Gondar, college of medicine and health sciences. Written informed consent was obtained from each study participants. Confidentiality and anonymity of the information was also maintained.

#### Operational definitions

*Male involvement* When partners’ of pregnant women attended both HIV counselling and testing (HCT) during ANC visit for the purpose of PMTCT of HIV service utilization [12].

*Knowledge about PMTCT* When respondents knew at least one way of PMTCT from three questions [19].

*Partners* Those male who had relation with woman that gave birth and or pregnant for him in the study period [19].

### Result

#### Socio-demographic characteristics of study participants

More than half 317 (56.5%) of the respondents were between the age range of 25–34 years with the mean age of 34.23 years old. Most of the participants 531 (94.6%) were married. Majority of the participants 376 (67.0%) and their partners 406 (72.4%) were attained secondary education and above (Table 1).

**Table 1:** Socio demographic characteristics of the study participants in Debre Tabor town, Northwest Ethiopia, 2018 (*n* = 561)

Variables	Frequency	Percentage
Age		
25–34	317	56.5
35–44	196	34.9
≥ 45	48	8.6
Religion		
Orthodox	539	96.1
Others^a^	22	3.9
Marital status		
Married	531	94.6
Single cohabitated	19	3.4
Separated	11	2.0
Ethnicity		
Amhara	554	98.8
Others^b^	7	1.2
Male educational status		
Not formal education	85	15.2
Primary education	100	17.8
Secondary and above	376	67.0
Partners’ educational status		
Not formal education	64	11.4
Primary education	91	16. 2
Secondary and above	406	72.4
Male occupation		
Self-employee	235	41.9
Government employee	326	58.1
Partners’ occupation		
Self-employee	302	53.8
Government employee	259	46.2
Monthly income in ETB		
Below 1000 birr	77	13.7
1000–1999	62	11.1
2000–2999	83	14.8
Above 3000	339	60.4

Key words: others^a^ **=** Muslim (2.9%), Catholic (0.9%) and protestant (0.2%). Others^b^ = Tigray (1.1%) and Oromo (0.2%)

#### Male partners’ reproductive history

Around a third 212 (37.8%) of male partners’ were pregnant and all of them were attend ANC. Most of them 482 (85.9%) have at least one child and almost all 468 (97.1%) were gave birth at heath institution (Table 2).

**Table 2:** Reproductive health characteristics of partners at Debre Tabor town, Northwest Ethiopia, 2018

Variables	Frequency	Percentage
Number of pregnancy		
One	229	40.8
More than one	332	59.2
Number of child		
None	79	14.1
1–4 children	469	83.6
5 and above	13	2.3
Last child age (year)		
≤ 1	357	74.1
> 1	125	25.9
Place of delivery		
Health institution	468	97.1
Home	14	2.9
Current pregnant status		
Yes	212	37.8
No	349	62.2
Number ANC		
Yes	212	100
No	0	0

#### Knowledge of the participant on PMTCT of HIV

Most of the participants 438 (78.1%) were responding PMTCT of HIV is used for protecting the baby getting HIV from mother. More than half of the study participants 333 (54%) were describing HIV can be transmitted from mother to child during pregnancy. Around a third of the participants weren’t go to PMTCT clinic.

#### Programmatic factors

Barriers of involving in PMTCT of HIV clinic service utilization were; lack of space to accommodate male 46 (8.2%), harsh language of health professionals 68 (12.1%), no availability of permanent PMTCT services 11 (2%), and long waiting time 52 (9.3%).

#### Factors associated with male involvement in PMTCT of HIV

In this study 119 (21.2%) of males (95%CI 17.8%, 24.8%) were involved in PMTCT of HIV. Being government employee (AOR = 3.73, 95%CI (2.169, 6.419)), partners’ who had ever heard about PMTCT (AOR = 2.46, 95%CI (1.20, 5.02)), discussed with their partners’ about PMTCT (AOR = 3.11, 95%CI (1.43, 6.55)), partners’ who were inform the need to go PMTCT clinic (AOR = 2.45, 95%CI (1.17, 5.14), health workers’ friendly approach (AOR = 2.36, 95%CI (1.34, 4.15)), and long waiting time (AOR = 0.36, 95%CI (0.216, 0.610)) were found to be significantly associated with male involvement in PMTCT of HIV (Table 3).

**Table 3:** Multivariable analysis of factors affecting male involvement in PMTCT of HIV in Debre Tabor town, Northwest Ethiopia, 2018 (n = 561)

Variables	Male involvement in PMTCT services utilization		COR (95%CI)	AOR (95%CI)	P-value
	Involved	Not involved			
Partner educational status					
Not formal education	8	77	1.00	1.00	1.00
Primary education	6	94	0.61 (0.21, 1.85)	0.57 (0.17, 1.95)	0.37
Secondary and above	105	271	3.73 (1.74,7.99)*	1.25 (0.50, 3.80)	0.64
Male educational status					
No formal education	5	59	1.00	1.00	1.00
Primary education	15	76	2.33 (0.80, 6.77)	0.96 (0.27, 3.32)	0.94
Secondary and above	99	307	3.8 (1.49, 9.75)*	0.75 (0.23, 2.46)	0.64
Partners occupational status					
Self-employee	21	214	1.00	1.00	1.00
Government employee	98	228	4.38 (2.38, 7.27)	3.73 (2.17, 6.42)**	*< 0.001*
Male occupational status					
Self-employee	54	248	1.00	1.00	1.00
Government employee	65	194	1.54 (1.02, 2.31)*	0.68 (0.38, 1.20)	0.18
Income per month					
Below 1000 birr	7	70	1.00	1.00	1.00
1000–1990 birr	5	57	0.88 (0.26, 2.91)	0.72 (0.19, 2.72)	0.62
2000–2999	14	69	2.03 (1.68, 8.52)	1.08 (0.35,3.37)	0.90
Above 3000 birr	93	246	3.78 (1.68, 8.52)*	1.20 (0.41, 3.33)	0.78
Go to ANC clinic with partner					
Yes	91	253	2.43 (1.53, 3.86)*	0.97 (0.55, 1.72)	0.92
No	28	189	1.00	1.00	1.00
Couples HCT					
Yes	96	257	3 (1.84, 4.92)*	1.5 (0.60, 3.80)	0.39
No	23	185	1.00	1.00	1.00
Ever heard about PMTCT					
Yes	11	144	4.74 (2.47,9.10)	2.46 (1.20, 5.02)**	*0.014*
No	108	298	1.00	1.00	1.00
Discussion about PMTCT with partner					
Yes	106	220	8.23 (4.49, 15.07)	3.11 (1.43, 6.55)**	*0.004*
No	13	222	1.00	1.00	1.00
Partners who were informed the need to go PMTCT clinic					
Yes	105	226	7.12 (3.98,12.91)	2.45 (1.17, 5.14)**	*0.017*
No	14	216	1.00	1.00	1.00
Health workers friendly approach					
Yes	98	217	4.84 (2.92, 8.03)	2.36 (1.34, 4.15)**	*0.003*
No	21	225	1.00	1.00	1.00
Long waiting time					
Yes	4	48	0.347 (0.16, 0.74)	0.35 (0.15, 0.79)**	*0.012*
No	115	394	1.00	1.00	1.00

NB 1.00= reference, * Statistically significant on bivariate analysis, ** Statistically significant on multivariable analysis

Significantly associated values are indicated in italic

### Discussion

The study result revealed that 21.2%, (95%CI 17.8%, 24.8%) of males were involved in PMTCT of HIV program. Our finding is comparable with a previous studies reported from Gondar town, North west Ethiopia, and Mekelle, Northern Ethiopia 20.9% and 20%, respectively [10, 11]. This implies that male participation in PMTCT of HIV is poor. However, the finding of this study is lower than with a previous studies reported in Arba Minich Town, Southern Ethiopia [12], and Addis Ababa City, Central Ethiopia [13]. The difference might be due to the inclusion criteria of the study population in both studies. In Arba Minch town, Southern Ethiopia, all male partners of reproductive age women who gave birth during the previous 1 year where as in Addis Ababa, central Ethiopia sampled male partners’ who were attending ANC/PMTCT services were included in the study population. In addition, the study setting, educational status, urbanization status, availability and accessibility of the service might be the difference on the service utilization. The finding of this study also lower than a previous studies conducted in Kenya, 31% [15], Thailand 46% [16], and Uganda 26% [17]. The difference also might be due to accessibility and availability of the service. In our study area there are few public and very limited private health institutions that provide PMTCT of HIV service. In contrast, the finding is higher than a study conducted in Mwanza District, Malawi which had 13.7% of males involved in PMTCT of HIV service [14]. The difference could be related to difference in study period, information education communication and behavioral change in communication, since male involvement on PMTCT of HIV services is increased time to time due to mass media and health care provider’s awareness creation effort.

In our study, men’s whose occupation were government employee were 3.7 times more likely to be involved in PMTCT of HIV as compared to self-employee (AOR = 3.73, 95%CI 2.169, 6.419). This finding is in line with a previous studies conducted at Addis Ababa, Ethiopia and Gondar, Ethiopia [10, 12]. This might be due to government employees are more likely educated, and the fact that they are more exposed to information and can able to understand the burden of the problem.

Those participants who had discussion about PMTCT of HIV with their wives had increased involvement of PMTCT of HIV by 3 times as compared to their counterparts (AOR = 3.11, 95%CI 1.43, 6.55). This finding is in line with studies reported in Southern Ethiopia, Northwest Ethiopia, and in Addis Ababa, Ethiopia [9, 13, 18]. The possible reason might be due to men having discussion with their wives about HIV/AIDS, and PMTCT will help to share information and increase males’ understanding to focus on and give attention about PMTCT program.

Programmatic factors such as waiting time at PMTCT of HIV service and health professional unfriendly approach were decrease males’ involvement in PMTCT of HIV. Long waiting time at PMTCT of HIV clinic were decrease males’ involvement in PMTCT of HIV program by 2.9 times than those who got the service timely (AOR = 0.348, 95%CI 0.1, 0.790). The finding is consistent with studies conducted in Cameron [19], and rural Western Kenya [10]. The difference could be related to the difference in patient flow proportional to health professionals, and availability of trained health professionals in PMTCT of HIV at ANC clinic. Health professionals friendly approach were 2.4 times more likely to increase male involvement in PMTCT of HIV service utilization than the counterpart (AOR = 2.358, 95%CI 1.34, 4.15). The finding is comparable with a study conducted in Zimbabwe and Sub-Saharan Africa [20, 21]. This might be due to good welcoming approach and respectful care will improve the service utilization.

Men’s who had ever heard about PMTCT were 2.5 times more likely involved on PMTCT of HIV services program as compared to those who had no information (AOR = 2.45, 95%CI 1.20, 5.02). This finding is in line with a study finding in Gondar, North Ethiopia [10]. The possible explanation might be having information about PMTCT of HIV will help to know the benefit of PMTCT of HIV programme for them as well as their new borns.

Males who were informed by their partners’ about the need to go with them and availability of PMTCT of HIV at ANC clinic were 2.5 times higher to be accompanied than their counterparts (AOR = 2.45, 95%CI 1.17, 5.14). This finding is consistent with a previous studies conducted at Mekele, Northern Ethiopia and Arba Minch town and Zuria Woreda, Southern Ethiopia [8, 11]. This could be due to the fact that their partners did not tell the need to go with them and utilize PMTCT services might failed to appreciate the importance of males’ involvement for prevention HIV infection from mother to child.

In this study males and their partners’ who attended secondary education and above were significantly associated in bivariable analysis but not in multivariable analysis. On the other hand, a study done in Arba Minch town and Arba Minch zuria woreda those who attended secondary education and above were four times more involved in PMTCT of HIV than the counterpart [8]. The difference could be related to the difference in the study area and period.

### Conclusion

The prevalence of male involvement in PMTCT of HIV at Debere Tabor Town was poor. Ever heard about PMTCT of HIV, discussed about PMTCT of HIV, female partner told the need to go PMTCT of HIV clinic, health workers friendly approach, being government employee and long waiting time were the determinate factors of male involvement in the PMTCT of HIV service. Improving service provision including respectful care and health education on PMTCT of HIV for males and their partner shall be emphasized by the government.

## Limitations of the study

Self-report might have introduced social desirability bias. Since, we have used cross sectional study design it is a poor predictor of cause and effect relationship.

## Supplementary information

**Additional file 1.** Data collection tool.

## Data Availability

The data set used in this study is available from the corresponding author and can be accessible through reasonable request.

## Abbreviations

AIDS: Acquired immune deficiency syndrome
ANC: Ante natal care
AOR: Adjusted odds ratio
CI: Confidence interval
COR: Crude odds ratio
HIV: Human immune deficiency virus
MTCT: Mother to child transmission
PMTCT: Prevention of mother to child transmission
