# Supplementary material for: Male involvement in prevention of mother to child transmission of human immuno virus and associated factors among partners’ of reproductive age women at Debre Tabor town, Northwest Ethiopia: a community based cross sectional study
Source: BMC Res Notes. 2020 Mar 30;13:190. doi: 10.1186/s13104-020-05023-3 (PMC7106685; doi:10.1186/s13104-020-05023-3)
Supplement: Supplementary file 1 — Additional file 1. Data collection tool. [file 13104_2020_5023_MOESM1_ESM.docx]

Instructions: use this questionnaire while you interview the participant. Encircle the possible answer (more than one answer is possible) and write the required responses in the spaces provided.

Part I**: Socio demographic characteristics**

| S.N. | | QUESTION | | ANSWER | | | | | | REMARK | | | | | |
| --- | --- | --- | --- | --- | --- | --- | --- | --- | --- | --- | --- | --- | --- | --- | --- |
| 101 | | How old are you? | | Age….. | | | | | | completed year | | | | | |
| 102 | | What is your religion? | | A. married B. Separated C. Single, cohabitating D. divorced E. widowed | | | | | |  | | | | | |
| 103 | | What is your current marital status? | | A. Orthodox B. Muslim  C. protestant D. catholic  E. other specify-------------------- | | | | | |  | | | | | |
| 104 | | What is your Ethnic group | | 1. Amhara 2. Oromo 3. Tigray 4. Other, specify….. | | | | | |  | | | | | |
| 105 | | Level of education? | | A. Unable to read and write  B. Read and write  C. Elementary  D. Secondary  E. college and above | | | | | |  | | | | | |
| 106 | | Female partner’s level of education? | | A. Unable to read and write  B. Read and write  C. Elementary  D. Secondary  E. college and above | | | | | |  | | | | | |
| 107 | | What is your occupation? | | A. Government employ  B. Merchant  C. Daily laborer  D. Student  E. Other specify | | | | | |  | | | | | |
| 108 | | What is your occupation? | | A. Government employ  B. Merchant  C. House wife  D. Student  E. Daily laborer  F. Others…. | | | | | |  | | | | | |
| 109 | | How much is your/family total monthly income? | | ----------- with EBR? | | | | | |  | | | | | |
| RART II: **Female partners reproductive history** | | | | | | | | | | | | | | | |
| 201 | | How many times did your partner become pregnant? | | A. 1 B. ≥2 | | | | | |  | | | | | |
| 202 | | How many children do you have? | | A. No child B. 1-4 C. 5+ | | | | | |  | | | | | |
| 203 | | How old is your last child | | 1. ≤ 1year B. >1year | | | | | |  | | | | | |
| 204 | | \| Where did she give birth give? \|  \| \| --- \| --- \| | | 1. Health institution B. Home | | | | | |  | | | | | |
| 205 | | Is your partner now pregnant? | | 1. Yes B. no | | | | | |  | | | | | |
| 206 | | If yes for Q5did she start ANC follow up? | | A .Yes B. No | | | | | |  | | | | | |
| Part III: **KNOWLEDGE OF PMTCT** | | | | | | | | | | | | | | | |
| 301 | Have you ever gone for antenatal clinic with your partner? | | | | 1. Yes--- B. no…. | | | |  |  |  |  |  |  |  |
| 302 | If no to question 301, what is reason/s for your answer? | | | | 1. Financial constraints 2. Lack of time due to work 3. It’s for women 4. Health workers attitude towards men 5. Time when the program is done not favorable 6. Others specify---------------------- | | | | Encircle all selected answers | |  |  |  |  |  |
| 303 | What is PMTCT? | | | | 1. Protecting the baby from getting HIV from mothers 2. Removing HIV from the baby 3. I do not know | | | | Encircle all selected answers | |  |  |  |  |  |
| 304 | When Can the virus that causes AIDS be transmitted from an HIV positive mother to her baby? | | | | 1. Pregnancy 2. Labor and delivery 3. Breast feeding 4. I do not know | | | | Encircle all the selected answers | |  |  |  |  |  |
| 305 | If pregnant mother is sero positive, do you know when does she start ART treatment? | | | | A .Yes, continue question 306 B. No | | | |  | |  |  |  |  |  |
| 306 | When does a pregnant woman start ARV treatment if she is sero positive? | | | | A. First trimester.   1. Second trimester. 2. Third trimester. 3. Any time after confirmed 4. I don’t know | | | | Encircle all the selected answers | | | | | |  |
| 307 | Do you know importance of going to PMTCT/ANC programs with partner? | | | | 1. Yes B. No, | | | |  |  |  |  |  |  |  |
| 308 | Would you accept couple counseling and testing of HIV during PMTCT program? | | | | | 1. Yes, go to part IV…. B. No… | |  |  |  |  |  |  |  |  |
| 309 | Give reasons why not accepting? | | | | | 1. Staff do not keep confidentiality 2. Staff members do not have counseling skills 3. Afraid because of my past sexual lifestyle 4. Already know my status 5. Others specify -- | | Encircle all selected answers | | | |  |  |  |  |
| PART IV SOURCES OF INFORMATION | | | | | |  | |  |  |  |  |  |  |  |  |
| 401 | Have you ever heard about PMTCT? | | | | | 1. Yes B. No | |  | | | | | |  |  |
| 402 | If yes to Q 1, what was your initial source of PMTCT knowledge? | | | | | A. Health workers  B. Mass media  C. your partner  *D. Others (specify)____* | | *encircle all that the participant mentions* | | | | | |  |  |
| 403 | Have you had a discussion on PMTCT With your partner? | | | | | A. Yes B. No | |  | | | | | |  |  |
| 404 | Did your partner tell you that you need to go for PMTCT program with her? | | | | | 1. Yes 2. No | |  |  |  |  |  |  |  |  |
| PART V PROGRAMATIC FACTORS | | | | | | | |  |  |  |  |  |  |  |  |
| 501 | Did health workers doing at ANC/PMTCT clinic provide services with friend line approach? | | | | | 1. Yes 2. No | |  |  |  |  |  |  |  |  |
| 502 | If No for question 2 what is/are the drawback/s? | | | | | 1. Lack of spaces to accommodate male partners in ANC/PMTCT clinic 2. Harsh language of skilled health professionals 3. No availability of permanent PMTCT services 4. Long duration of waiting time to get services 5. If *others (specify)____* | | *encircle all that the participant mentions* | | | | |  |  |  |
|  | PART V*I* MEN INVOLVEMENT IN PMTCT/ANC | | | | | | |  |  |  |  |  |  |  |  |
| 601 | Did you know your wife’s appointment for ANC when she is/ was pregnant? | | A.YES B.NO | | | | |  |  |  |  |  |  |  |  |
| 602 | Did you discuss with your wife counseling and testing for HIV the when she is/was pregnant? | | A.YES B.NO | | | | |  |  |  |  |  |  |  |  |
| 603 | Have you ever gone together with your wife to an ANC/PMTCT clinic? | | A.YES B.NO … | | | | |  |  |  |  |  |  |  |  |
| 604 | Have you ever counseled and tested for HIV together with your wife at an ANC/PMTCT clinic? | | 1. Yes…. B. No… | | | |  |  |  |  |  |  |  |  |  |
| 605 | Did you support your wife’s antenatal visits financially? | | Yes…. B. No… | | | |  |  |  |  |  |  |  |  |  |
| 606 | Do you accept if health professionals inform you to use condom during the time of your wife’s pregnancy?? | | Yes…. B. No… | | | |  |  |  |  |  |  |  |  |  |

Thank you
